# Progress towards universal HIV testing among TB patients in Viet Nam: a retrospective cohort evaluation of TB/HIV surveillance, 2011–2017

**DOI:** 10.1186/s40249-019-0536-6

**Published:** 2019-04-01

**Authors:** Nguyen Binh Hoa, Nguyen Viet Nhung

**Affiliations:** 1 Viet Nam National Lung Hospital, National Tuberculosis Programme Viet Nam, 463 Hoang Hoa Tham street, Badinh District, Hanoi, Viet Nam; 2 Centre for Operational Research, International Union Against Tuberculosis and Lung Disease, Paris, France; 3 Hanoi Medical University, Hanoi, Viet Nam

**Keywords:** Tuberculosis, HIV, Trend, Viet Nam

## Abstract

**Background:**

Tuberculosis (TB) and HIV remain a major causes of morbidity and mortality globally. We conducted an analysis of TB/HIV surveillance data to describe the trends in HIV testing coverage and HIV positivity rate among TB patients in Viet Nam, 2011–2017.

**Main text:**

This was a descriptive study based on review and analysis of surveillance data from the National Tuberculosis Control Programme from 2011 to 2017. During this period, 721 342 TB cases were diagnosed. Of these, 520 490 (72.2%) had a previously documented HIV status or were tested for HIV during TB care and treatment. The proportion of TB patients whose HIV status was reported increased, from 58.5% in 2011 to 82.9% in 2017 (*P* value for trend = 0.014). The proportion of TB patients infected with HIV decreased, from 8.0% in 2011 to 3.7% in 2017 (*P* value for trend = 0.018).

**Conclusions:**

The proportion of TB patients with a reported HIV status was increased from 2011 to 2017, however HIV testing coverage remained below the National Tuberculosis Control Programme targets (≥ 90%). National Tuberculosis Control Programme needs to focus on ensuring every registered TB patients has a documented HIV status, ensuring full coverage of HIV testing as part of routine TB care.

**Electronic supplementary material:**

The online version of this article (10.1186/s40249-019-0536-6) contains supplementary material, which is available to authorized users.

## Multilingual abstracts

Please see Additional file 1 for translations of the abstract into the five official working languages of the United Nations.

## Background

Tuberculosis (TB) remains a major cause of morbidity and mortality globally [1]. In 2017, World Health Organization (WHO) estimated there were 1.3 million deaths from TB among persons without HIV, and an additional 300 000 deaths from TB among people living with HIV. An estimated, 10 million people fell ill with TB, equivalent to 133 per 100 000 population globally, with 9% of incident TB cases among people living with HIV [1]. Globally, among the 6.4 million TB cases notified in 2017, 60% were reported to have a documented HIV status in 2017 and coverage in the WHO Western Pacific Region was lower at 51% [1].

Viet Nam ranks 15th among the 30 highest-TB burden countries in the world [2]. WHO estimated Viet Nam had 124 000 incident TB cases in 2017, equivalent to an incidence rate of 129 per 100 000 population. There were 4500 TB/HIV cases, making up 3.6% of all TB cases. WHO also estimated there were 12 000 TB deaths, including 840 among people living with HIV in Viet Nam, 2017 [1].

HIV is strongest known risk factor for developing TB and TB is also the leading cause of death among people living with HIV [3]. WHO has issued recommendations on TB/HIV collaborative activities, including: (1) Establish and strengthen the mechanisms for delivering integrated TB and HIV services; (2) Reduce the burden of TB among people living with HIV and initiate early antiretroviral therapy (ART); (3) Reduce the burden of HIV in patients with presumptive and diagnosed TB [4] In response, the Viet Nam Ministry of Health, National Tuberculosis Control Programme (NTP) and HIV Control Programme (VAAC) issued guidelines for collaborative activities. HIV counselling and testing of TB patients was first implemented with donor support in 2005, among 26 high HIV burden provinces. These activities were expanded and in 2009, when the NTP guidelines were updated to require HIV testing for all TB patients to ensure early HIV diagnosis and referral for HIV care and treatment. In 2015, the standard national TB register and surveillance system was also revised to include HIV status and ART status per WHO guidelines. In 2016, the TB register was further updated to distinguish between TB patients with pre-existing documentation of HIV status and those whose HIV status was first ascertained during TB treatment (by the time of treatment outcome). Since 2011, TB and HIV programs have coordinated procurement of HIV test kits for on-site HIV testing at TB registration and treatment sites with donor support and implemented the program in all provinces of Viet Nam. We describe the annual trend in national coverage of known HIV status among registered TB patients and the proportion of TB patients with a documented HIV-positive status over a 7 year period, from 2011 to 2017, using TB surveillance data.

## Methods

Routine NTP TB/HIV surveillance data from all 63 provinces were reviewed and analysed. The design was a retrospective observational cohort study describing the annual number and proportion of registered TB patients with a documented HIV status and the number and proportion of TB patients reported to have an HIV positive status, from 2011 to 2017.

### Data source

The Viet Nam TB Information Management Electronic System (VITIMES) is a web-based system designed to collect patient-level data on all registered TB cases, including their HIV status, per WHO guidelines [4]. Each district TB unit enters both required aggregate indicator data as well as individual case-based data for each TB patient documented in the standard paper-based TB Register. The standard indicators include the number and proportion of registered TB cases with a documented HIV status and the test results.

### Data analysis

Data were exported from VITIMES to Microsoft Excel (Excel) and analysed using both Excel 2010 and Stata version 14 (Stata Corp LLC, 4905 Lakeway college station, Texas, USA). The proportion of TB patients with a known HIV status was calculated by taking the number of TB patients with an HIV test result recorded in the TB register and dividing by the total TB cases registered during the same period. The proportion of TB patients with a positive HIV test was calculated by taking the number of TB patients with a positive HIV test result and dividing by the number of all TB patients with an HIV test result. We used nptrend test (Cuzick test) for assessment the trend of non-parametric data. Point estimates are shown with 95% confidence intervals for proportions. A *P*-value < 0.05 was regarded as statistically significant. Trends in HIV testing and coverage were further analysed at the subnational level, stratified by the eight Viet Nam government-defined socio-economic regions of Viet Nam.

### Ethics

This paper was based on an analysis of routine collected surveillance data from the electronic NTP register, VITIMES. Program leadership and staff led the review, analysis and interpretation of the data with formal NTP permission.

## Findings

During the period of 2011–2017, 721 342 TB patients were diagnosed and registered for TB treatment. Of these, 520 490 TB patients (72.2%) had a documented HIV test result at the time of registration or were tested for HIV during TB care and treatment. Over time, the proportions of TB patients with a documented HIV status increased from 58.5% in 2011 to 82.9% in 2017 (*P* = 0.014), see Table 1.

**Table 1:** Proportion of TB patients with a documented HIV test result and the proportion with a positive test result, Viet Nam, 2011–2017

Year	Total TB cases (*n*)	TB cases with a documented HIV status (*n*)	Percentage	TB/HIV positive (*n*)	Percentage (95% *CI*)
2011	100 535	58 819	58.5	4714	8.0 (7.8–8.2)
2012	103 812	66 141	63.7	4531	6.9 (6.7–7.0)
2013	100 721	70 417	69.9	4301	6.1 (5.9–6.3)
2014	102 087	74 092	72.6	3875	5.2 (5.1–5.4)
2015	102 676	79 979	77.9	3438	4.3 (4.2–4.4)
2016	105 839	83 467	78.9	2936	3.5 (3.4–3.6)
2017	105 672	87 575	82.9	3234	3.7 (3.6–3.8)
2011–2017	721 342	520 490	72.2	27 029	5.2 (5.1–5.3)

*TB* Tuberculosis, *CI* Confidence interval, *HIV* Human immunodeficiency virus

Among the 520 490 TB patients with a documented HIV test during this period, 27 029 (5.2, 95% *CI*: 5.1–5.3%) had a positive HIV test result. The proportion of people living with HIV among TB patients with known HIV status decreased, from 8.0% in 2011 to 3.7% in 2017 (*P* = 0.018).

**Fig. 1:**
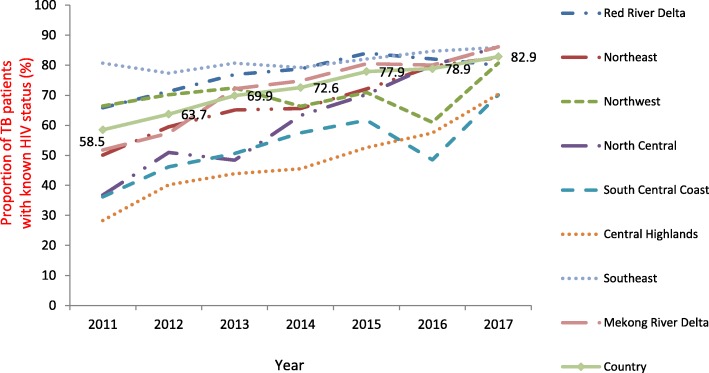
Proportions of HIV testing among tuberculosis patients, by 8 socio-economic regions, Viet Nam, 2011–2017. HIV: Human immunodeficiency virus. TB: Tuberculosis

HIV testing coverage and HIV positivity rate trends from 2011 to 2017, stratified by the eight socio-economic regions are shown in Fig. 1. The trend in the proportion of TB patients with a known HIV status significantly increased in every region (*P* < 0.05), except the North-West region (*P* = 0.662) and South Central Coast region (*P* = 0.054).

**Fig. 2:**
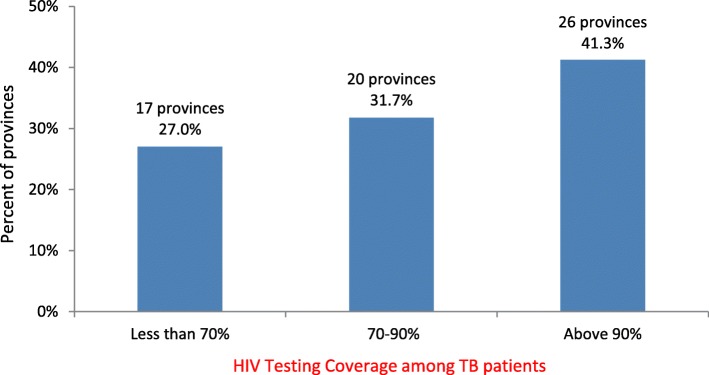
Distribution of provinces by proportions of HIV testing among tuberculsosis patients, Viet Nam, 2017. HIV: Human immunodeficiency virus. TB: Tuberculosis

Figure 2 presents the distribution of provinces with different levels of HIV testing coverage among TB patients in 2017, categorized as less than 70, 70 to 90%, and above 90%. Of 63 provinces in Viet Nam, 17 provinces (27%) had a low HIV testing coverage of less than 70%. An additional 20 (31.7%) had an HIV testing coverage of 70–90% and 26 provinces (41.3%) met the national goal of ≥90%. HIV testing coverage among TB patients was lowest in Vinh Phuc province (25.8%) and highest in Nam Dinh and Vinh Long provinces (99%).

**Fig. 3:**
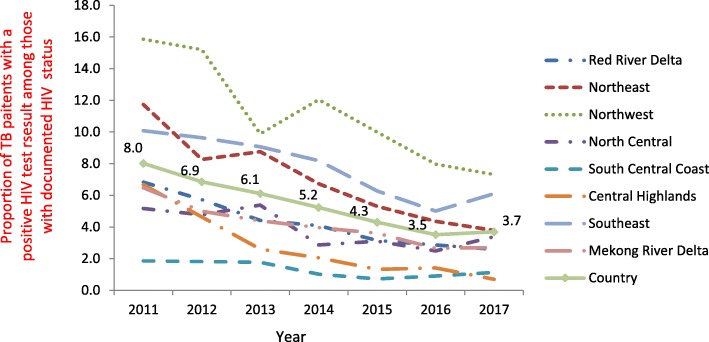
Proportions of HIV positive among tested tuberculosis patients, by 8 socio-economic regions, Viet Nam, 2011–2017. HIV: Human immunodeficiency virus. TB: Tuberculosis

Figure 3 shows the HIV positivity rate among TB patients with a documented HIV test result, classified by socio-economic region. The HIV positivity rate among TB patients decreased in all regions (*P* < 0.05), except in the North Central region (*P* = 0.015) and South Central Coast region (*P* = 0.066).

**Fig. 4:**
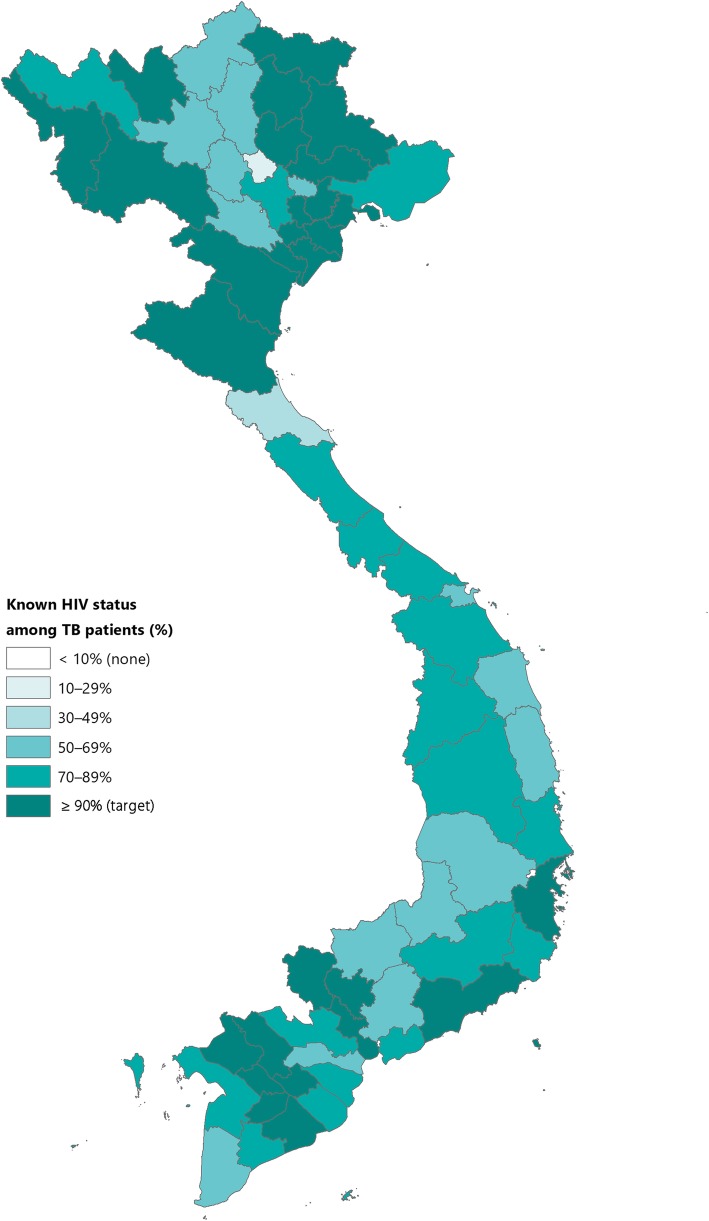
Proportion of tuberculsosis patients with a documented HIV test result by provinces in Viet Nam, 2017. HIV: Human immunodeficiency virus. TB: Tuberculosis

**Fig. 5:**
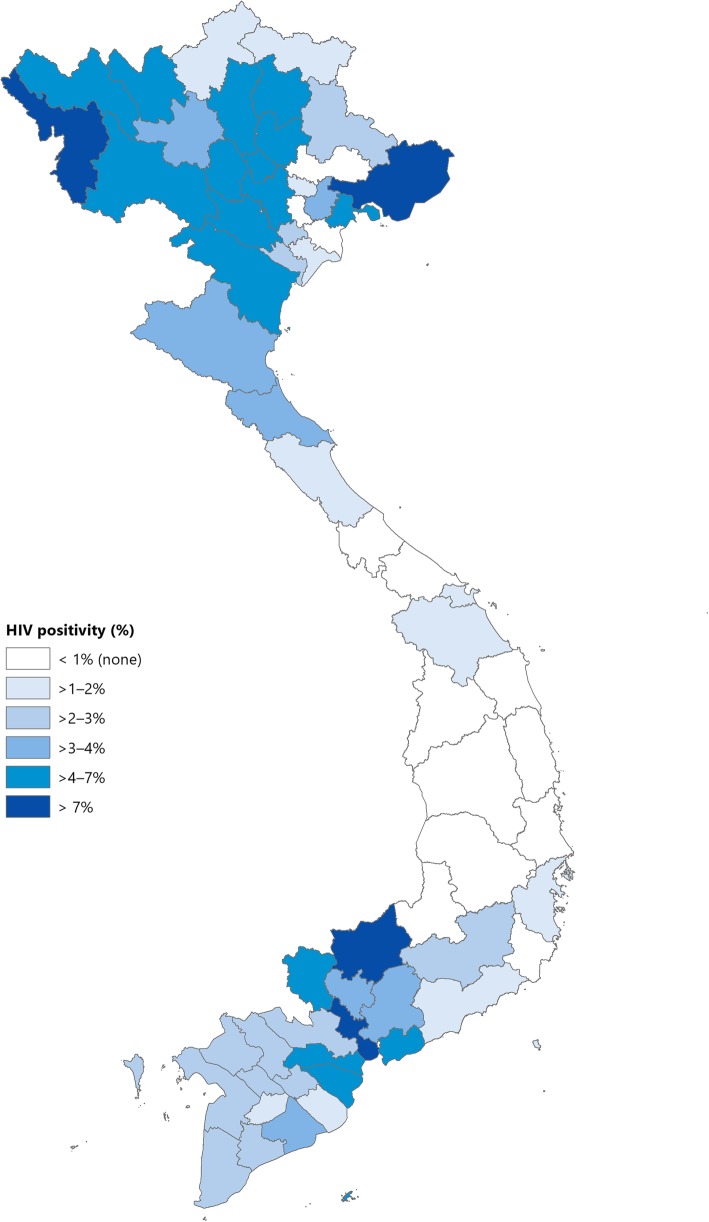
Proportion of tuberculsosis patients with a positive HIV test result by provinces in Viet Nam, 2017. HIV: Human immunodeficiency virus. TB: Tuberculosis

The geographic variation in HIV testing coverage among TB patients, HIV positivity rate and burden of HIV by province in 2017 is illustrated in Figs. 4 and 5. Among the 63 provinces in Viet Nam, only 26 (41%) currently meet the national target of ≥90% of TB patients have a documented HIV status. Among the remaining 37 provinces with HIV testing coverage rates below 90% the coverage ranged between 25.8 and 89.3%.

## Discussion

As the coverage of HIV status documentation among TB patients increased from 2011 to 2017, the HIV positivity rate among TB patients has declined. During the early years, it is possible that HIV testing was more likely to be based on clinician-judgement and identification of individuals at high risk of HIV exposure. With the increasing adoption of and access to universal HIV testing for all TB patients, the proportion with an HIV-positive status may have decreased because of the eventual inclusion of relatively more TB patients with a lower risk of HIV exposure. Even so, the HIV positivity rate among TB patients, which has stabilized between 3 and 4% in recent years, is substantially higher than that of the general population (< 1%) underscoring the importance of ascertaining the HIV status of all TB patients in Viet Nam and linking persons living with HIV rapidly to care and treatment. During this same period, access to anti-retroviral treatment for people living with HIV also increased from an estimated 26% in 2011 to 50% in 2017 [5]. It is possible that increased access to anti-retrovirals reduced the risk of active TB resulting in fewer people living with HIV developing tuberculosis annually [6]. While this might be true, the risk of developing TB and death from TB continues to be much higher among people living with HIV even on treatment than for persons without HIV thus all TB/HIV collaborative efforts remain program imperatives. WHO estimates corroborate the absolute decline in TB/HIV case seen in the surveillance trends, citing lower numbers of TB/HIV cases in Viet Nam over time, from an estimated 14 000 (11 000–18 000) in 2011 to 4500 (3700–5400) in 2017 [2, 7].

The NTP surveillance systems are now able to provide a more robust national population estimate given over 80% of TB patients have a documented HIV status reported [8]. Based on these data, WHO estimated 4.0% of TB patients had an HIV positive status in 2017 which is similar to the NTP estimate [1]. Viet Nam reported higher HIV testing coverage in 2017 compared to the global coverage (60% of notified cases) and the WHO Western Pacific Region coverage (50% of notified cases). However, HIV testing coverage is still lower than some other countries in the Greater Mekong sub-region such as Cambodia (87%) and Myanmar (90%).

WHO recommendations call for provision of HIV counselling and testing to persons with diagnosed TB or presumptive TB [4, 9, 10]. Currently, the Viet Nam NTP policy only requires provision of HIV testing for diagnosed and registered TB patients. Initial pilots of testing presumptive TB patients are underway to assess yield and feasibility in the setting of a concentrated HIV epidemic like Viet Nam.

The main limitation of this observational report is that it is based on a retrospective analysis of routinely reported programmatic data which raises the potential concern about data quality. However, a national review of TB programme data led by WHO in 2013 included an assessment of the TB surveillance system using a WHO checklist of standards and benchmarks and concluded that the TB surveillance system in Viet Nam was well-functioning with many strengths [11, 12]. The data available for analysis in this report is based on the aggregate indicator reports only. The transition from aggregate indicator reporting to a case-based electronic register is still underway. Thus a detailed exploration of individual demographic and clinical factors associated with a TB patient knowing their HIV status and factors associated with TB/HIV could not be conducted. Nevertheless, the reported descriptive trends document program progress and challenges. Another limitation is that we did not reported ART coverage among TB patients with a positive HIV test result in this analysis.

## Conclusions

In conclusion, the proportion of TB patients with a known HIV status has increased from 2011 to 2017, however it is still lower than the national target of ≥90%. Viet Nam NTP should continue to focus on providing universal HIV testing access to TB patients in all provinces, especially in the 37 provinces where the HIV testing coverage below 90%. Importantly the patients identified to be living with HIV while accessing TB services should be promptly referred to HIV treatment to ensure the best patient outcomes.

## Additional file

Additional file 1: Multilingual abstracts in the five official working languages of the United Nations. (PDF 338 kb)

## Abbreviations

ARV: Antiretroviral
HIV: Human immunodeficiency virus
NTP: National Tuberculosis Programme
TB: Tuberculosis
VAAC: Viet Nam Administration of HIV/AIDS Control
WHO: World Health Organization
