# Supplementary material for: Progress towards universal HIV testing among TB patients in Viet Nam: a retrospective cohort evaluation of TB/HIV surveillance, 2011–2017
Source: Infect Dis Poverty. 2019 Apr 1;8:25. doi: 10.1186/s40249-019-0536-6 (PMC6442430; doi:10.1186/s40249-019-0536-6)
Supplement: Supplementary file 1 — Multilingual abstracts in the five official working languages of the United Nations. (PDF 338 kb) [file 40249_2019_536_MOESM1_ESM.pdf]

Translation of the abstract into the five official working languages of the United Nations

التقدم المحرز نحو اختبار فيروس نقص المناعة البشرية الشامل بين مرضى السل في فييت نام: تقييم استعراضي لمراقبة السل / فيروس نقص المناعة البشرية 2011–2017

Nguyen Binh Hoa and Nguyen Viet Nhung

ملخص

الخلفية: لا يزال السل وفيروس نقص المناعة البشرية من الأسباب الرئيسية للمراضة والوفيات على مستوى العالم. أجرينا تحليلاً لبيانات ترصد السل / فيروس نقص المناعة البشرية لوصف الاتجاهات في تغطية اختبار فيروس نقص المناعة البشرية ومعدل إيجابية فيروس نقص المناعة البشرية بين مرضى السل في فيتنام ، 2011 - 2017.

النص الرئيسي: كانت هذه دراسة وصفية مبنية على مراجعة وتحليل بيانات المراقبة من البرنامج الوطني لمكافحة السل في فترة 2011 إلى 2017.وخلال هذه الفترة، تم استعراض وتقييم وإقفال 117 قضية. ومن بين هؤلاء ، كان 520 490 (72.2٪) مصابًا بحالة فيروس نقص المناعة البشرية موثقة مسبقًا أو تم اختبارهم لفيروس نقص المناعة البشرية أثناء رعاية مرضى السل وعلاجهم. ارتفعت نسبة مرضى السل الذين تم الإبلاغ عن حالة الإصابة بفيروس نقص المناعة البشرية ، من 58.5 ٪ في عام 2011 إلى 82.9 ٪ في عام 2017 (P لقيمة الاتجاه = 0.014). ارتفعت نسبة مرضى السل الذين تم الإبلاغ عن حالة الإصابة بفيروس نقص المناعة البشرية ، من 8.0 ٪ في عام 2011 إلى 3.7 ٪ في عام 2017 (P لقيمة الاتجاه = 0.018).

لاستنتاجات: تم زيادة نسبة مرضى السل الذين تم الإبلاغ عن إصابتهم بفيروس نقص المناعة البشرية من عام 2011 إلى عام 2017 ، ومع ذلك ظلت تغطية اختبار فيروس نقص المناعة البشرية أقل من أهداف برنامج مكافحة السل الوطني (≥ 90 ٪). يحتاج البرنامج الوطني لمكافحة السل إلى التركيز على ضمان أن كل مرضى السل المسجلين لديهم حالة موثقة من الإصابة بفيروس نقص المناعة البشرية ، مما يضمن التغطية الكاملة لاختبار فيروس نقص المناعة البشرية كجزء من الرعاية الروتينية لمكافحة السل.

Translated from English version into Arabic by Aalya Al-Beeshi, revised by Ebtihal Ziyadah, through

TRANSLATORS
WITHOUT BORDERS

越南结核病患者中的艾滋病毒普查进展：2011 年至 2017 年结核病/艾滋病监测数据的回顾性队列评估

Nguyen Binh Hoa and Nguyen Viet Nhung

摘要

引言：结核病（TB）和人类免疫缺陷病毒（HIV）仍是全球发病率和死亡率的一个重要诱因。本研究对越南 TB/ HIV 的监测数据进行了分析，以描述 2011–2017 年结核病患者中的艾滋病检测覆盖率和艾滋病毒阳性率的趋势。

正文：基于 2011 年至 2017 年国家结核病控制规划监测数据，本研究对其进行了描述性回顾分析。在此期间，共诊断出 721 342 例 TB。其中，520 490（72.2%）TB 患者有艾滋病记录，或在 TB 治疗期间接受过 HIV 检测。据报道，在 TB 患者中，记录并报道 HIV 状态

比例从 2011 年的 58.5%增长到 2017 年的 82.9％（趋势 *P* 值为 0.014）；HIV 感染者比例从 2011 年的 8.0％下降到 2017 年的 3.7％（趋势 *P* 值为 0.018）。
结论：研究表明，在 2011 年至 2017 年间，越南 TB 患者中的 HIV 感染报告的比例有所增加，但 HIV 检测覆盖率仍低于国家结核病控制规划目标（≥90％）。 国家结核病控制规划需要确保每个登记的 TB 患者都有 HIV 感染状况报告，确保所有 TB 患者接受 HIV 检测，并作为 TB 常规治疗的内容。

Translated from English version into Chinese by Qing-Yun Chen, revised by Jin Chen

Des progrès vers le dépistage universel du VIH parmi les patients tuberculeux au Vietnam : évaluation rétrospective de la surveillance de cohortes TB/VIH, 2011-2017

Nguyen Binh Hoa et Nguyen Viet Nhung

RÉSUMÉ

Contexte : La tuberculose (TB) et le VIH restent des causes majeures de morbidité et de mortalité dans le monde entier. Nous avons analysé des données de surveillance de ces deux maladies au Vietnam de 2011 à 2017, afin de décrire les tendances de la couverture par le dépistage du VIH et le taux de positivité au VIH parmi les patients tuberculeux.
Discussion : Cette étude descriptive s'appuie sur la revue et l'analyse des données de surveillance du programme national vietnamien de lutte contre la tuberculose de 2011 à 2017. Au cours de cette période, 721 342 cas de tuberculose ont été diagnostiqués. Parmi ceux-ci, 520 490 (72,2 %) avaient déjà fait l'objet d'un test de VIH ou ont été testés dans le cadre de la prise en charge de la tuberculose. La proportion de patients tuberculeux dont le statut VIH était rapporté a augmenté de 58,5 % en 2011 à 82,9 % en 2017 (valeur de *P* pour la tendance = 0,014). La proportion de patients tuberculeux infectés par le VIH a diminué de 8,0 % en 2011 à 3,7 % en 2017 (valeur de *P* pour la tendance = 0,018).
Conclusions : Bien que la proportion de patients tuberculeux dont le statut VIH était rapporté ait augmenté entre 2011 et 2017, la couverture par le dépistage du VIH est restée en dessous des objectifs du programme national de lutte contre la tuberculose (≥ 90 %). Dans le cadre de ce programme, il importe de faire en sorte que le statut VIH de chaque patient tuberculeux enregistré soit documenté, afin d'assurer une couverture complète par le dépistage du VIH dans le cadre du traitement de routine de la tuberculose.

Translated from English version into French by Suzanne Assenat, revised by Gwenaelle Le Jan-Moulart, through

Прогресс в отношении всеобщего тестирования на ВИЧ среди больных туберкулезом во Вьетнаме: ретроспективная когортная оценка наблюдения за туберкулезом / ВИЧ, 2011 г.-2017 г.

Нгуен Бинь Хоа (Nguyen Binh Hoa) и Нгуен Вьет Нхунг (Nguyen Viet Nhung)

РЕФЕРАТ

Справочная информация: Туберкулез (ТБ) и ВИЧ остаются основными причинами заболеваемости и смертности во всем мире. Мы провели анализ данных из ТБ/ВИЧ наблюдений, чтобы описать тенденции в охвате тестирований на ВИЧ и уровень ВИЧ положительных пациентов среди больных ТБ во Вьетнаме в 2011–2017 гг.

Основной текст: Было проведено описательное исследование, основанное на оценке и анализе данных наблюдений, полученных от Национальной программы по борьбе с туберкулезом в 2011-2017 гг. За этот период было диагностировано 721342 случая заболеваемости туберкулезом. Из них 520490 (72,2 %) случая ранее имели зарегистрированный статус ВИЧ или сдавали анализы на ВИЧ во время ТБ терапии. Доля больных ТБ с зарегистрированным ВИЧ статусом выросла с 58,5 % в 2011 году до 82,9 % в 2017 году (*P-значение* для тренда = 0,014). Доля больных туберкулезом, инфицированных ВИЧ, снизилась с 8,0 % в 2011 году до 3,7 % в 2017 году (*P-значение* для тренда = 0,018).

Выводы: Доля больных ТБ с зарегистрированным ВИЧ-статусом увеличилась с 2011 по 2017 год, однако тестирования на ВИЧ оставались ниже плановых показателей Национальной программы по борьбе с туберкулезом (≥ 90 %). Национальная программа по борьбе с туберкулезом должна сосредоточиться на обеспечении каждого больного ТБ зарегистрированным ВИЧ статусом и повсеместном проведении тестирований на ВИЧ как части обычной процедуры при лечении ТБ.

Translated from English version into Russian by by Gunel Huseynbayova, revised by Michael Orlov, through

TRANSLATORS WITHOUT BORDERS

Avances hacia la universalización de las pruebas de detección de VIH en pacientes con TB en Vietnam: un estudio de cohortes retrospectivo de la supervisión de TB/VIH, 2011–2017

Nguyen Binh Hoa and Nguyen Viet Nhung

RESUMEN

Antecedentes: La tuberculosis (TB) y el VIH continúan siendo una de las causas principales de morbilidad y mortalidad a nivel mundial. Se realizó un análisis de los datos de vigilancia de TB/VIH

para describir las tendencias en la cobertura de las pruebas de VIH, y la tasa de positivos de VIH entre los pacientes con tuberculosis en Vietnam durante el período 2011-2017.

Texto principal: Se trataba de un estudio descriptivo basado en la revisión y análisis de los datos de vigilancia del Programa Nacional de Control de la Tuberculosis entre 2011 y 2017. Durante este período se diagnosticaron 721.342 casos de TB. De éstos, 520.490 pacientes (72,2%) tenían una condición de VIH previamente informada, o fueron sometidos a pruebas de VIH durante el cuidado y tratamiento de la TB. La proporción de los pacientes con TB con posible seropositividad documentada aumentó del 58,5 % en el 2011 al 82,9 % en el 2017 (el valor de *p* para la tendencia = 0,014). La proporción de pacientes con TB infectados con VIH disminuyó del 8,0 % en el 2011 al 3,7 % en el 2017 (el valor de *p* para la tendencia = 0,018).

Conclusiones: La proporción de pacientes con TB con una condición de VIH registrada aumentó del 2011 al 2017. Sin embargo, la cobertura de las pruebas de VIH se mantuvo por debajo de los objetivos del Programa Nacional del Control de la Tuberculosis (≥ 90 %). El Programa Nacional del Control de la Tuberculosis debe centrarse en garantizar que cada uno de los pacientes registrados con TB tenga documentada su condición de VIH, garantizando la cobertura integral de las pruebas de VIH como parte de la atención rutinaria de la TB.

Translated from English version into Spanish by Celia Martinez, revised by J. Manuel Bas y Mansilla, through

TRANSLATORS
WITHOUT BORDERS
